# Bowel function after anterior rectal resection for cancer: short and long-term prospective evaluation with low anterior rectal syndrome (LARS) score in a cohort of Cameroonian patients

**DOI:** 10.11604/pamj.2024.47.171.32287

**Published:** 2024-04-09

**Authors:** Guy Aristide Bang, Georges Roger Bwelle Moto, Joseph Cyrille Chopkeng Ngoumfe, Eric Patrick Savom, Marcelin Ngowe Ngowe

**Affiliations:** 1 Department of Surgery and Specialties, Faculty of Medicine and Biomedical Sciences, University of Yaoundé I, Yaoundé, Cameroon,; 2 Surgical Unit, Yaoundé University Teaching Hospital, Yaoundé, Cameroon,; 3 Digestive Surgical Unit, Yaoundé Central Hospital, Yaoundé, Cameroon

**Keywords:** Anterior rectal resection, rectal cancer, bowel dysfunction, low anterior rectal syndrome (LARS), Cameroon

## Abstract

**Introduction:**

bowel dysfunction is the most common and disabling complication after anterior rectal resection (ARR) for cancer. We aimed to evaluate these complications in a cohort of Cameroonian patients, using the low anterior rectal syndrome (LARS) score.

**Methods:**

we conducted a descriptive and analytical cross-sectional study, in two university hospitals of Yaoundé (Cameroon). Prospectively, we collected the records of all patients aged at least 18 years who had an ARR indicated for rectal cancer from January 2015 to March 2018. Alive patients among them were subsequently received in consultation at 1 and 3 years after surgery, for short and long-term assessment of their digestive function using the LARS score.

**Results:**

during the study period, 28 patients underwent anterior rectal resection for rectal cancers. Short-term bowel function was evaluated in 23 patients. Their mean age was 48.42 ± 12.2 years and 14 were males. LARS was present in 10 of them (43.47%) and classified as “minor” in the majority of cases (n=6). The commonest bowel dysfunction at this term was splitting of stool (56.53%). Long-term digestive function was evaluated in 11 patients; LARS was found in 3 of them (27,27%) and classified as minor in all cases. Perfect continence was significantly improved (p=0.003) in the long term compared to the short-term status. Continence (p=0.049) and urgency (p=0.048) were better in patients who had a low colorectal anastomosis compared to those who had a colo supra-anal anastomosis.

**Conclusion:**

after ARR for cancer, there is a high prevalence of LARS in the short term with an improvement in the long term.

## Introduction

Colorectal cancer (CRC) is the third most common cancer worldwide, with approximately 1,500,000 new cases per year [1]. Although their incidence is decreasing in the West, it is increasing in Africa, with a clear predominance in young adults [2].

The evolution of neo-adjuvant therapies as well as surgical techniques has allowed more and more anterior rectal resections (ARR) to be performed instead of abdominoperineal amputations [3]. However, ARR is associated with significant morbidity. This morbidity is represented by anastomotic fistulas, pelvic collections, tumour recurrence and bowel dysfunction [3,4].

Bowel dysfunction is the most common and disabling complication after ARR [5-7]. They may include: total or selective incontinence for flatus and/or faeces, urgency, clustering, and emptying difficulties [8]. These functional digestive sequelae can be grouped under the term low anterior resection syndrome (LARS) [9]. LARS is related to the removal of the rectal reservoir during proctectomy and the extensive carcinological dissection in a limited pelvic space [6]. In our context, ARR is often accompanied by extensive colonic resection because of the high prevalence of locally advanced forms [10], which may increase the digestive sequelae.

Studies show that 60-90% of patients suffer from LARS [7,11] which has a negative impact on their quality of life (QoL). Although LARS improves during the first two years following surgery, it persists beyond this period in nearly 60% of patients; one in two patients will have severe symptoms [12,13].

In Cameroon, although the QoL of patients after rectal cancer surgery has been assessed as good overall [14], bowel function after rectal cancer surgery has not been studied. The aim of the present study was to evaluate bowel function with LARS score, in the short and long term, in a cohort of Cameroonian patients who underwent ARR.

## Methods

**Study design:** we conducted a descriptive and analytical cohort study in two university hospitals in Yaoundé (capital of Cameroon): Yaoundé Central Hospital and Yaoundé University Teaching Hospital.

**Setting:** the choice of these two hospitals is justified by the fact that they have the largest activity of adult digestive oncology surgery in the city of Yaoundé.

**Participants:** prospectively, we collected the records of all patients aged at least 18 years who had an ARR indicated for rectal cancer from January 2015 to March 2018 (i.e. 39 months). Alive patients among them were subsequently received in consultation at 1 and 3 years after surgery, for short and long-term assessment of their digestive function respectively. This time frame was calculated from the date of primary resection in patients who did not have a stoma or from the date of stoma closure in patients who had one.

**Data sources/measurement:** the LARS score was used to assess the digestive functional outcome of patients [15]. The items of the LARS Score (Annex 1) are incontinence for flatus or for liquid stool, frequency of bowel movements, clustering of stools, and imperiousness. It allows the categorization of patients into three groups: no LARS (0-20 points), minor LARS (21-29 points), and major LARS (30-42 points). An interview was conducted by one of the investigators to evaluate the bowel function according to the LARS score. If necessary, some items were explained to the participants in the local language.

**Variables:** other variables studied were: age and sex, tumour data, and therapeutic data (neo-adjuvant and adjuvant treatment, manual or mechanical anastomosis, protective enterostomy).

**Statistical methods:** data were recorded using CS Pro version 6 software and analysed using IBM_ SPSS (Statistical Package of Social Sciences), version 20.0. Quantitative variables were expressed as mean with standard deviation. Comparison of proportions was done using the Chi-square test for qualitative variables and the Mann-Whitney test for quantitative variables. Cox logistic regression was used to identify the factors associated with LARS, and their hazard ratio (HR), the difference being considered statistically significant for a p-value ≤0.05.

Informed consent was obtained from each patient before inclusion in the study. Ethical clearance was obtained from the ethics and research committee of the Faculty of Medicine and Biomedical Sciences of the University of Yaoundé I. Study authorizations were obtained from the administrative services of the various health facilities.

## Results

**Participants:** during our study period, 28 patients underwent ARR for rectal tumours, with an annual incidence of 8.4. Five died within 12 months of surgery (17.8%).

**Descriptive and outcome data:** we were then able to assess LARS in the short term for 23 patients. There were 14 male and 9 female patients, with a sex ratio of 1.55. Their mean age was 48.42 ± 12.2 years with extremes ranging from 25 to 80 years. The majority of tumours were located in the lower rectum (n=10, 43.47%). Tumours of the middle rectum (n=7) represented 30.43%, and tumours of the upper rectum (n=6), 26.08% of cases. According to the tumour, node and metastasis (TNM) classification, 21.73% of the tumours were stage I (n=5), 17.39% stage II (n=4) and 60.86% stage III (n=14). The anastomoses were performed with a circular mechanical stapler in 30.43% of cases (n=7) and manually in 69.56% (n=16). A bypass stoma to protect the anastomosis was performed in each case. This was a terminal colostomy in 82.60% of cases (n=19) and a lateral colostomy in 17.39% of cases (n=4). The anastomoses were colo supra-anal (n=17, 73.91%) and low colo-rectal (n=6, 26.08%). None of these patients had received radiotherapy. All anastomoses were performed without a colonic pouch.

**Main results:** the evaluation of the short-term functional result in these 23 patients revealed the existence of a LARS in 43.48% of them (n=10). It was minor in 26.08% (n=6) and major in 17.39% (n=4). Perfect continence was found in 56.52% of patients (n=13). The majority of patients (n=18, 78.26%) had a normal defecation frequency. Table 1 shows the short-term functional results.

**Table 1:** short-term functional digestive outcomes

Digestive function	N	Percent	P
**Gas leaks**			
No	12	52.17%	0.05
Less than 1 time/week	7	30.43%	
More than 1 time/week	4	17.39%	
**Liquid stool leakage**			
No	13	56.52%	0.06
Less than 1 time/week	6	26.08%	
More than 1 time/week	4	17.39%	
**Stool frequency /24h**			
More than 7	0	0	0.52
4 to 7 per day	5	21.73%	0.45
1 to 3 per day	18	78.26%	
Less than 1 per day	0	0	
**Splitting of stool in 1 hour**			
No	10	43.47%	0.07
Less than 1 time/week	4	17.39%	
More than 1 time/week	9	39.13%	
**Urgency (imperiousness)**			
No	16	69.56%	0.46
Less than 1 time/week	5	21.73%	
More than 1 time/week	2	8.69%	
Total	23	100%	

Long-term functional outcomes (3 years) could be assessed in 11 patients, the other 12 had died. LARS syndrome was found in 3 patients (27.27%) and was minor in all cases. Anal continence was perfect in 9 patients (81.81%). Long-term defecation frequency was normal in all patients. The long-term functional results are shown in Table 2.

**Table 2:** long-term functional digestive outcomes

Digestive function	N	Percent	P
**Gas leaks**			
No	8	72.72%	
Less than 1 time/week	3	27.27%	0.041
More than 1 time/week	0	0	
**Liquid stool leakage**			
No	9	81.81%	
Less than 1 time/week	2	18.18%	0.056
More than 1 time/week	0	0	
**Stool frequency / 24h**			
More than 7	0	0	
4 to 7 per day	0	0	0.4
1 to 3 per day	11	100%	
Less than 1 per day	0	0	
**Splitting of stool in 1 hour**			
No	8	72.72%	
Less than 1 time/week	2	18.18%	0.06
More than 1 time/week	1	9.09%	
**Urgency (imperiousness)**			
No	9	81.81%	
Less than 1 time/week	2	18.18%	0.032
More than 1 time/week	0	0	
Total	11	100%	

Of all the elements of digestive function, only perfect continence was significantly improved (p=0.003) in the long term compared to the short-term situation (Table 3). The anastomotic height had a statistically significant influence on perfect continence and urgency (Table 4). So, continence (p=0.049) and urgency (p=0.048) were better in patients who had a low colorectal anastomosis compared to those who had a colo supra-anal anastomosis.

**Table 3:** digestive functional results in relation to time after anastomosis

	Delay after anastomosis				P
Digestive function	Short-term		Long-term		
	N	Percent	N	Percent	
**Perfect continence**					
Yes	13	56.52%	9	81.81%	0.003
No	10	42.47%	2	18.18%	
**Polyexoneration**					
Yes	5	21.73%	0	0	0.07
No	18	78.36%	11	100%	
**Stool splitting**					
Yes	13	56.52%	3	27.27%	0.67
No	10	42.47%	8	72.72%	
**Urgency (imperiousness)**					
Yes	7	30.43%	2	18.18%	
No	16	69.56%	9	81.81%	0.048
Total	23	100%	11	100%	

**Table 4:** short-term digestive function in relation to the level of anastomosis

	Distance from the anastomosis to the anal margin				P
Digestive function	Colo sup-anale (3-6cm)		Low colorectal (> à 6 cm)		
	N	Percent	N	Percent	
**Perfect continence**					
Yes	8	47.05%	5	83.33%	0.049
No	9	52.94%	1	16.66%	
**Polyexoneration**					
Yes	5	29.41%	0	0	0.61
No	12	70.58%	6	100%	
**Stool splitting**					
Yes	9	52.95%	1	16.66%	0.51
No	8	47.05%	5	83.33%	
**Urgency (imperiousness)**					
Oui	7	41.17%	0	0	0.048
Non	10	58.82%	6	100%	
Total	17	100%	6	100%	

## Discussion

The objective of this study was to assess bowel function after ARR for rectal cancer in a cohort of Cameroonian patients. With the LARS score, we found that 43.48% and 27.27% of patients had a bowel dysfunction in the short and long term, respectively.

Defecation disorders, ranging from incontinence to difficult evacuation, occur commonly after sphincter-sparing surgery for rectal cancer [5-7,11]. Patients undergoing sphincter-saving operations may develop a number of unpleasant symptoms, typically faecal soiling and urgency [16]; this explains the poorer quality of life of patients undergoing anterior resection than those of patients with abdominoperineal extirpation reported in some studies [17,18]. In addition to resection of the rectum, factors such as age, sex, anastomotic height, technique of reconstruction, radiotherapy, and anastomotic leakage have been identified to influence the severity of functional sequelae [19,20]. The evaluation of bowel function after anterior resection is then important.

Since its description in 2012 [9,21], the LARS Score has become one of the major tools in the evaluation of bowel function after anterior rectal resection for cancer and has been translated into 30 languages [15,22-26]. Unfortunately, this score is little used in Africa and to the best of our knowledge, this is the first African study to use it.

In the short term (one year after stoma closure), we found a high prevalence of LARS in our patients (43.48%), thus confirming the high frequency of digestive disorders after ARR. These disorders were mostly of the “minor” type in the majority of cases (6 out of 10). In Western series, patients most often present in the short-term with a major LARS. We believe that one possible explanation for this is the greater use of radiotherapy for rectal cancer in the West. The standard treatment for rectal cancer includes a neoadjuvant chemoradiotherapy [27]. The deleterious effects of radiotherapy on digestive function have already been demonstrated [28,29]. In our series, no patient received radiotherapy. In fact, our country has only one radiotherapy centre, located in a different city from the one where our study took place. Distance, long waiting times and costs are factors that limit the accessibility of our patients to radiotherapy. Patients who had an anastomosis within 3 cm of the anal margin had poorer continence than others (p=0.049). Indeed, the closer the tumour dissection is to the sphincters, the greater is the risk of damaging them. We believe that the use of mechanical staplers for low rectal anastomosis may help to reduce the incidence of incontinence. In fact, continence after manual anastomosis seems to be less than after anastomosis with mechanic staplers [30]. In our series, only 30.43% of these anastomoses were performed mechanically.

In the short term, perfect continence was found in 56.52% of our patients. Many authors report perfect continence in the short term in between 65 and 95% of cases [31-33]. The absence of a colonic pouch in our series could explain this difference. Indeed, the use of a colonic J-pouch has been widely described as improving the functional results of digestion in numerous studies [29,34]. The use of a colonic J-pouch could also decrease the prevalence of stool fragmentation found in 56.52% of our patients. Indeed, Barrier *et al*. [31] found a proportion of 49% of stool fragmentation in patients who had a direct anastomosis compared to 31% in those in whom a pouch had been made.

The assessment of long-term bowel function was biased by the large number of deaths between surgery and postoperative year 3. However, we found that the prevalence of LARS decreased over time from 43.48% to 27.27% between postoperative years 1 and 3. This study confirms the improvement of bowel function over time after ARR [31,35]. It is therefore important to explain it to the patient, especially about continence. In this report, continence improved significantly between the short and long term.

Other factors influencing functional outcome have been described in the literature, notably the extent of rectal resection. So, the digestive function would be almost normal in 92 to 100% of patients if more than 5 cm of rectum remained above the anastomosis [36] and impaired if less than 5 cm remained.

**Limitations:** they include: a small sample size, absence of data on the management of patients with bowel dysfunction, and non-assessment of the association between LARS and QoL. However, the prospective evaluation, especially in the long term, and the use of the LARS score are the main strengths of this report. We encourage African practitioners to use this score in the follow-up of their patients after ARR for cancer.

## Conclusion

Bowel function after anterior rectal resection in our context is characterized by a high prevalence of LARS among patients in the short term. However, there is an improvement over time, with a smaller prevalence in the long term. In the majority of cases, dysfunctions are classified as “minor” according to the LARS score. Bowel function could be improved by the realization of a colonic J-pouch.

### What is known about this topic

- *Digestive function is impaired after anterior rectal resection;*
- *The LARS Score is used to assess this digestive function, which improves over time*.

### What this study adds

- *Digestive function results after anterior rectal resection for rectal cancer in Cameroon;*
- *The use of the LARS Score as a reference measurement tool for digestive function*.

## Annex

**Annex 1**: low anterior rectal syndrome (LARS) score scoring instructions (PDF - 93Kb)
